# Social Identity Complexity, Corporate Social Responsibility, and Brand Love of Multiple Leagues in Professional Sport

**DOI:** 10.3389/fpsyg.2022.861656

**Published:** 2022-03-25

**Authors:** Chanwook Do, Natasha T. Brison, Juho Park, Hyun-Woo Lee

**Affiliations:** Department of Health and Kinesiology, Texas A&M University, College Station, TX, United States

**Keywords:** social identity complexity, tolerance, corporate social responsibility, brand authenticity, brand love, sport league experience

## Abstract

How can corporate social responsibility initiatives influence brand love? Based on the theory of social identity complexity, we examined whether greater complexity of a sport fan’s multiple identifications with sport leagues led to higher multicultural tolerance and more positive perceptions of leagues’ corporate social responsibility activities. Further, brand authenticity was tested as a variable intervening between perceived corporate social responsibility and brand love. We analyzed this serial mediation effect impacting sport fans’ brand love for their multiple, favored and less favored, sport leagues. Participants (*N* = 242 Amazon Mechanical Turk workers) answered the scale item questionnaire for model assessment. The hypothesized model was supported as the indirect effect through all mediators was significant (43.42% of total indirect effects). Our results suggest that when sport fans acknowledge a high overlap among league fan groups (low social identity complexity), their tolerance is more likely to be higher than those who have a low overlap. Such high levels of tolerance influence how fans perceive corporate social responsibility initiatives, and these effects build up for fans to perceive the brand to be more authentic (i.e., based on their continuity, credibility, integrity, and symbolism). These antecedents affected brand love through a serial mediation. Sport league managers should consider the diverse aspects across leagues (i.e., different fan characteristics, media operations, game schedules) for harmonious coexistence with other leagues (e.g., by collaborating on promotions and reducing overlap of schedules) and maintain brand authenticity for their social initiatives to result in a greater brand love in the consumer’s mind.

## Introduction

Professional sport leagues compete and collaborate to retain existing and acquire new customers. While most sports fans follow multiple sports (McDonald et al., 2010), how they develop and maintain their trust and love toward the game and the league can depend on how fans perceive each league’s communications and publicity efforts (Giulianotti, 2002; de Oliveira et al., 2021). As Manthiou et al. (2018) noted, brand authenticity and brand love are imperative indicators of a successful brand management as it reflects the trustworthiness and psychological connection with a brand. Considering the ongoing integrated marketing communications as branding efforts of professional leagues, therefore, it is imperative to understand how sport consumers’ individual differences and preferences in professional leagues can affect how they perceive the branding activities of professional leagues. One aspect of fan characteristics can be demonstrated by their profiles in which leagues they choose to follow.

Sport consumer researchers highlight the role of psychological attachments and, in particular, the practical significance of social identification with sport organizations (Heere et al., 2011; James et al., 2019). Nonetheless, the notion that multiple identities coexist and how the overlap among group identities influence individual perception is an under-studied issue (Brewer and Pierce, 2005; Delia, 2015; Lee et al., 2020). Further, Brewer and Pierce underscored the predictive effect of identity overlap on individuals’ tolerance to recognize and respect diversity. Understanding how fans follow multiple sport leagues and how it affects their perceptions of their favored or unfavored league warrants particular attention when sport fans are known to follow multiple sports (Cobbs et al., 2017). As fans would have varying degrees of identification toward multiple leagues, such identity complexity would affect individuals’ ability to identify and appreciate the social values the league holds and promotes. In this study, we investigate how fans’ overlapping identifications with multiple leagues are related to their tolerance, which affects their evaluation of a league’s social responsibility initiatives, authenticity, and brand love.

We highlight the variables examined in the current study by reviewing the literature on social identity complexity and brand authenticity. By highlighting the serial mediation effects of tolerance, perception of social initiatives, and authenticity, we attempt to address the gap in branding and social identity research in the professional sport context by examining the role of three mediators. Direct and indirect effects are dissected further to discuss implications to scholars and brand managers.

## Literature Review

Brand expansion for increasing market share and market growth is dependent on how many consumers are attracted to and willing to share the brand’s identity. With this regard, people are more engaged with brands that demonstrate consistent and credible behaviors in responding to consumers’ requests (Ahuvia, 2005), leading to demonstrating a more relatable brand identity (Manthiou et al., 2018). Likewise, sport leagues attempt to capture the attention of consumers by showing authentic behavior, not only through matches on the playing field but also via various marketing communication campaigns (Joo et al., 2019). As authentic efforts to connect with their fans, for example, leagues have led campaigns to increase ball-in-play time (e.g., penalizing long delays of play such as intervals of goal kicks in soccer and between pitches in baseball) and reduce commercial time to provide better service to the fans (e.g., improving the continuity of broadcasting). Furthermore, authentic efforts include movements aimed to better communicate the integrity of the league such as implementing community service programs to be socially responsible and improving players’ safety. These efforts can address the complaints from players and fans (e.g., the league is commercialized too much), as well as enhance the psychological connections between leagues and customers (James et al., 2002; Williams et al., 2005).

While sport organizations continuously seek new marketing plans and communication tools to catch fans’ attention (Tsiotsou, 2013), the use of Corporate Social Responsibility (CSR) programs—contributing to societal goals—has been actively adopted in the sport industry. For example, all four major leagues have adopted the pink breast cancer ribbon campaign and are promoting local community initiatives in their hometowns to engage and expand their fan base (Walker and Heere, 2011; Alhouti et al., 2016). Further, leagues in the United States (US) took a unified stance in protesting social discrimination and left a strong impression on their fans (Evans et al., 2020). To accomplish the goals of CSR, which is developing brand identity through societal community development, sport organizations need to view marketing initiatives through the eyes of new and existing customers to gain positive fan attitudes and brand images (Babiak and Wolfe, 2006; Walker and Kent, 2009).

Despite the fact that sport fans following multiple leagues (Cobbs et al., 2017), scholars within the field of sport management have conducted little research on sport fans’ multiple identifications at the league level. Using the theory of social identity complexity (Roccas and Brewer, 2002), Brewer and Pierce (2005) assessed people’s identity complexity by measuring their tolerance and emotion when accepting out-group members. Specific to sport management, Meyer (2014) studied the relationship between fans’ multiple identifications and their tolerance, and Lee et al. (2020) conducted a study on the association between fans’ team identification and their place identification.

However, previous studies in sport management lack an explanatory model for the relationship between social identity complexity and brand authenticity. As such, researchers have called for more research on identity complexity and brand authenticity in sport management (e.g., Heere et al., 2011; Delia, 2015; Kucharska et al., 2020). In terms of social identity complexity, Brewer and Pierce (2005) found that people who have a sport identity showed less tolerant behavior compared to those who do not. Furthermore, Meyer (2014) revealed sport fans have varying degrees of tolerance related to in-group members accepting members from an out-group. Nevertheless, many studies do not consider the evaluation of the organization when accepting out-group members. Considering social identity complexity and tolerance as dispositions of individual characteristics to embrace diversity, such disposition is likely to influence people’s recognition and respect to social initiatives and their emotional connections with sport leagues.

### Professional Sport League’s Brand Research

Sport brands attract fans by using names, symbols, and associated meanings, and were evaluated based on the perceptions of consumers and sales or shares in the marketplace (Aaker, 1991). In the case of brand authenticity, scholars acknowledge the enormous worth of professional sport leagues as brands, but few researchers have concentrated on the brand authenticity of the league in the field of sport management. In order to increase the market value of professional sport leagues in the US, including the National Football League (NFL), the National Basketball Association (NBA), the National Hockey League (NHL), and Major League Baseball (MLB), which are valued at over $32.23 billion (Willis, 2020), a study on how brand authenticity entices consumers is needed (Morhart et al., 2015).

The relationships among the brands of the teams and leagues can be described by the concept of sport brand architecture (Kunkel et al., 2013). Sport brand architecture is segmented into three theoretical groups: league dominant, team dominant, and codominant (Kunkel et al., 2013). A league dominant architecture is illustrated by consumers who have a primary interest in the league brand and therefore may prefer to be more involved with activities at the league level than being involved with individual teams currently in the league (Kunkel et al., 2013). In the event that a professional sport team relocated from their home field to another city, fans who supported the team usually ascribe a nostalgic characteristic to the team (Kulczycki and Hyatt, 2005). For example, fans of a relocated team (e.g., the NFL team San Diego Chargers moving to Los Angeles in 2017 to become the Los Angeles Chargers) have a high possibility of becoming a fan of a new sport team in the league (Lock et al., 2009).

Team dominance is a fan’s perception that the value of a given team brand is relatively higher than the value of the league brand (Kunkel et al., 2013). Unique features (e.g., players, coaches, distinguished atmospheres) of the team can influence customers to reinforce team dominance (Kolbe and James, 2000). Team dominance is found in world-famous clubs, such as the Manchester United F.C., New York Yankees, and Los Angeles Lakers, all of which generally retain strong brand value, have a remarkable history and sell out for most games (cf. Deloitte, 2011).

In the case of a codominant architecture, consumers are equally interested in the brand of the league and a specific team (Kunkel et al., 2013). Based on such tendencies, consumers not only watch various matches within the league via media but also purchase several teams’ merchandise. For instance, fans who follow their favorite NFL team also watch other teams’ games on special days such as Monday Night Football (Kunkel et al., 2013). However, not many studies have focused on the evaluation of the league brand based on fans’ tolerance and external factors, including CSR, brand authenticity, and brand love. In particular, to better understand sport consumers’ perceptions toward sport league brands, it is imperative to investigate how identification with multiple leagues coexist in a consumer’s mind and how leagues’ efforts that are external to the match affects their connection with the league. We review these concepts and constructs in the following sections.

### Social Identity Complexity and Tolerance

Social identity complexity (Roccas and Brewer, 2002) is relatively a newer theoretical construct. This theory assesses the perceived degree of overlap among elements of a person’s multi-group membership. When the level of perceived overlap is high (i.e., low complexity), members of a group perceive other groups as being less different and follow more coherent behavioral outcomes (e.g., in-group favoritism and out-group discrimination) (Roccas and Brewer, 2002). Additionally, Brewer and Pierce (2005) conducted empirical research demonstrating that people’s attitudes can be changed, depending on the degree of tolerance by out-groups and diversity. For example, people with high identity complexity tend to be more accepting and tolerant of out-group members positively.

Scholars concur that research on social identity complexity is still in its early stages (Brewer and Pierce, 2005; Heere et al., 2011). Furthermore, among studies on sport management, views of multiple identities in sport are not in agreement. Brewer and Pierce (2005) found that sport fans tend to have not only a high overlap (i.e., low complexity) but also a lack of distinctiveness, suggesting that sport fans are a relatively homogeneous group compared to people who do not identify with sport. However, Meyer (2014) demonstrated that low overlap exists when only sampling sport fans. To extend these findings, Lee et al. (2020) studied sport fans to find an interaction between team identification and place identification on attendance intention.

Although all of these studies measured complexity using various factors (e.g., personal group, team identification and national identity, and team identification and place identification), complexity between professional sport leagues have not been considered. Each league in the US has unique characteristics (e.g., season start date, rules). Furthermore, the leagues do not consider other sport leagues as barriers when devising marketing plans. For instance, the NFL and the MLB advertised their own games during the NBA play-off in 2020 (e.g., NBA, 2020). Through this method, leagues are presented with an opportunity to attract other leagues’ fans and foster a multiple league identity among fans. Additionally, identity complexity can have positive effects on out-group members and fans’ purchase intentions (Brewer and Pierce, 2005; Lee et al., 2020). However, there is insufficient research on the association between fans’ identity complexity across leagues and perceived brand love. Therefore, the first hypothesis is:

H1. Sport fans’ identity complexity positively influences their brand love of the league.

Additionally, tolerance may interact with the level of identity complexity to influence people’s behavior. Brewer and Pierce (2005) found that the association between participants’ identity complexity and emotion toward the out-group depended on the degree of tolerance. Although this study assessed in-group members’ identity complexity in relation to emotions about out-group members, there is limited research about how in-group members evaluate their organizations when organizations accept out-group members and how levels of tolerance may affect their perceptions of a league. Therefore, the second hypothesis is:

H2. The level of fans’ tolerance mediates the effect of their identity complexity on their brand love for the league.

### Corporate Social Responsibility, Brand Authenticity, and Brand Love

Corporate social responsibility is the active behavior of an organization that takes responsibility for the environment and social welfare (Russo and Perrini, 2010). The importance of CSR initiatives has been emphasized due to their ability to showcase an organization’s image to the public (Babiak and Wolfe, 2006). To achieve a high evaluation of a brand, the company must demonstrate both uniqueness and conformity to CSR initiatives (Johansen and Nielsen, 2012). In the case of US professional sport leagues, leagues have their own CSR programs such as the construction of new infrastructures for a local community or offering job opportunities (Ahmed, 1991). Through CSR initiatives, people who do not have a particular league identity also can develop an interest in the league (Maignan and Ferrell, 2004). Despite the widespread nature of CSR programs across leagues that contribute to developing community and expanding the fan base, scholars in sport management have mostly conducted CSR research on specific teams (e.g., Walker and Kent, 2009; Baena, 2018). Research on specific teams has demonstrated that CSR programs not only facilitate the formation of fans’ identification (He and Li, 2011) but also enhance brand love for the league (Baena, 2018). CSR may mediate the effects of fans’ complexity of identities within the sport league on their brand love for the league. Therefore, the third hypothesis is:

H3. Perceived league’s CSR mediates the effect of fans’ identity complexity on their brand love for the league.

To determine the effect of brand authenticity, several scholars in the field of marketing have developed an objective scale that includes multiple dimensions (Napoli et al., 2014; Morhart et al., 2015). For instance, Morhart et al. (2015) proposed four dimensions of brand authenticity: history, credibility, integrity, and symbolism. In the case of Napoli et al. (2014), researchers similarly contended there are three categories of brand authenticity including heritage, sincerity, and quality commitment. When measuring brand authenticity through customer perceptions, it is imperative to understand the antecedents and consequences involving the perception process. For instance, Safeer et al. (2020) argued that brands perceived with high levels of brand authenticity bring optimal opportunities for not only engaging customers into celebrating the brand identity but also to create a positive image of the organization in the consumer’s mind. Through the evaluation of multidimensional brand authenticity characteristics, customers with higher levels of perceived brand authenticity may have higher love for the brand (Manthiou et al., 2018). In this, identity complexity is considered as a personal disposition affecting the perception process as an antecedent. Therefore, the fourth hypothesis is:

H4. A league’s brand authenticity mediates the effect of fans’ identity complexity on their brand love for the league.

**FIGURE 1:**
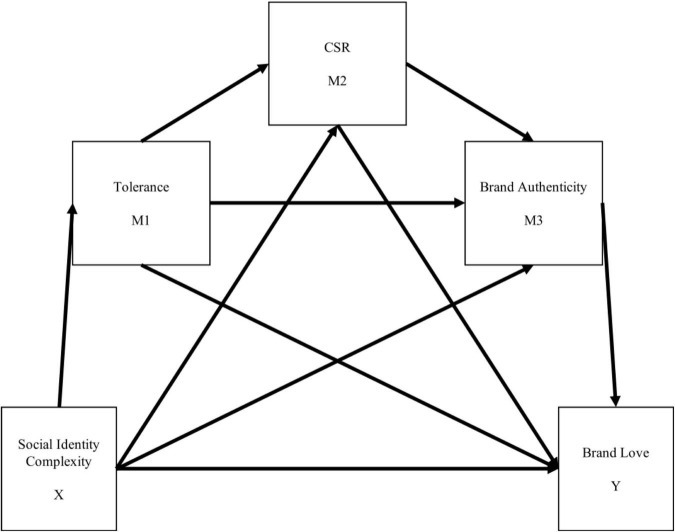
Conceptual model for this research.

Taking all of the above into account, sport fans’ identity complexity with multiple leagues and their tolerance are hypothesized as individual characteristics affecting value-laden CSR initiatives. CSR conveys the image of a brand to the local community (Babiak and Wolfe, 2006). In holding an image that is consistent with CSR, a brand has to demonstrate authentic behavior (Joo et al., 2019). As fans’ perceptions of CSR initiatives influence how they think of the brand’s authenticity, the proposed model consists of the serial mediations involving tolerance, CSR, and brand authenticity between league identity complexity and brand love (Figure 1). Hence, the following hypothesis is proposed:

H5. The levels of fans’ tolerance, the league’s CSR, and their perceived brand authenticity sequentially mediate the effect of fans’ identity complexity and their brand love for the league.

## Materials and Methods

### Participants

Participants for this study were recruited through Amazon’s Mechanical Turk (MTurk). As MTurk facilitates data collection from diverse participants who adequately represent the online-using population, the platform has been widely employed for research purposes (e.g., Mason and Suri, 2012; Larkin and Fink, 2016). To collect quantitative data, MTurk participants with a qualifying Human Intelligence Task (HIT) approval rate greater than 95% were included in this survey. The purpose of setting up a high HIT approval rate is to enhance research reliability (Hunt and Scheetz, 2019). On the basis of this condition, 270 respondents were recruited. After excluding 28 participants who did not pass the screening questions or had incomplete responses, 242 participants were included in the analysis.

### Measure and Procedures

Participants were first presented with an informed consent form. Those who agreed to participate in the study and signed the form were re-directed to the external questionnaire via Qualtrics. Participants rated their sport identity complexity across four major leagues in the US based on Brewer and Pierce’s (2005) scale (from 0 to 10 points). For example, participants were asked about the number of members overlapping across each league. Then, participants completed a series of tolerance-related questions (National Opinion Research Center, 1998) to assess their attitudes about various social issues on a seven-point Likert-type scale from 1 (strongly disagree) to 7 (strongly agree). Participants also ranked their favorite US professional sport leagues from top to bottom. Based on this ranking, we measured their perceptions of favorite and least favorite leagues’ CSR initiatives (Walker and Heere, 2011), brand authenticity (Morhart et al., 2015), and brand love (Carroll and Ahuvia, 2006) on a Likert scale from 1 (strongly agree) to 7 (strongly disagree). Finally, participants reported demographic information. Participants were compensated 50 cents for completing the 15-min questionnaire (see Appendix 1).

### Data Analysis

Data were coded using SPSS version 25 (IBM Corp, 2017). Before running the main analyses, all measures were evaluated using Cronbach’s alpha (Cronbach, 1951). In alignment with recommended values for research purposes (Nunnally, 1994), Cronbach’s alphas of 0.70 or above were regarded as acceptable. However, tolerance variables showed insufficient reliability, which has been reported in previous studies (Brewer and Pierce, 2005; Meyer, 2014). While Meyer (2014) used two variables for multiculturalism, the current study employed a single item of multiculturalism asking their perception of racial and ethnic groups maintaining their distinct customs and traditions. The single item considers respecting other cultures and was deemed relevant for measuring tolerance toward different cultures.

To test the hypotheses, mediation analyses with bootstrapping using Mplus 8 (Muthén and Muthén, 2017) were employed with fans’ league identity complexity as an independent variable, the evaluation of league brand love as a dependent variable, and the degree of tolerance, CSR, and brand authenticity as mediators. Two separate mediation analyses were conducted for favorite league and least favorite league.

## Results

Among the 242 final participants, 181 were male (74.8%) and 61 were female (25.2%). Ages ranged from 20 to 70 (*M* = 32.7, *SD* = 9.14). With respect to race and ethnicity, most participants were White (*n* = 98, 40.5%) followed by Asian (*n* = 79, 32.6%), Hispanic (*n* = 25, 10.3%), Native American (*n* = 21, 8.7%), African American (*n* = 18, 7.4%), and other (*n* = 1, 0.4%). Over a half of participants had a bachelor’s degree (*n* = 153, 63.2%). The NBA was the most favored league (*n* = 87, 36.0%) followed by the NFL (*n* = 76, 31.4%), the NHL (*n* = 43, 17.8%), and MLB (*n* = 36, 14.9%), while the NHL was least favored league (*n* = 78, 32.2%) followed by MLB (*n* = 73, 30.2%), the NBA (*n* = 46, 19.0%), and the NFL (*n* = 45, 18.6%). Tables 1, 2 displays the demographic characteristics of the participants.

**TABLE 1:** Demographic Characteristics of Sample (*n* = 242).

Variables	*n*	%
**Gender**		
Male	181	74.8
Female	61	25.2
**Age (*M* = 32.4, *SD* = 9.03)**		
20–29 years	99	40.9
30–39 years	109	45.0
40 years and older	34	14.0
**Race**		
Native American	21	8.7
Asian	79	32.6
African American	18	7.4
Hispanic	25	10.3
White	98	40.5
Other	1	0.4
**Education**		
High School Diploma	21	8.7
Associate Degree	19	7.9
University Degree In Progress	8	3.3
Bachelor’s Degree	153	63.2
Master’s Degree	39	16.1
Doctoral Degree	2	0.8

**TABLE 2:** Rankings for favorite and least favorite league.

		Ranking		
Favorite league (1st)		2nd	3rd	4th
NBA (*n* = 87)	NFL	45 (51.7%)	22 (25.3%)	20 (23.0%)
	NHL	22 (25.3%)	31 (35.6%)	34 (39.1%)
	MLB	20 (23.0%)	34 (39.1%)	33 (37.9%)
NFL (*n* = 76)	NBA	45 (59.2%)	12 (15.8%)	19 (25.0%)
	NHL	14 (18.4%)	34 (44.7)	28 (36.8%)
	MLB	17 (22.4%)	30 (39.5%)	29 (38.2%)
NHL (*n* = 43)	NBA	23 (53.5%)	5 (11.6%)	15 (34.9%)
	NFL	10 (23.3%)	16 (37.2%)	17 (39.5%)
	MLB	10 (23.3%)	22 (51.2%)	11 (25.6%)
MLB (*n* = 36)	NBA	19 (52.8%)	5 (13.9%)	12 (33.3%)
	NFL	9 (25.0%)	19 (52.8%)	8 (22.2%)
	NHL	8 (22.2%)	12 (33.3%)	16 (44.4%)

**Least favorite league (4th)**		**3rd**	**2nd**	**1st**

NBA (*n* = 46)	NFL	11 (23.9%)	16 (34.8%)	19 (41.3%)
	NHL	19 (41.3%)	12 (26.1%)	15 (32.6%)
	MLB	16 (34.8%)	18 (39.1%)	12 (26.1%)
NFL (*n* = 45)	NBA	7 (15.6%)	18 (40.0%)	20 (44.4%)
	NHL	13 (28.9%)	15 (33.3%)	17 (37.8%)
	MLB	25 (55.6%)	12 (26.7%)	8 (17.8%)
NHL (*n* = 78)	NBA	7 (9.0%)	37 (47.4%)	34 (43.6%)
	NFL	26 (33.3%)	24 (30.8%)	28 (35.9%)
	MLB	45 (57.7%)	17 (21.8%)	16 (20.5%)
MLB (*n* = 73)	NBA	8 (11.0%)	32 (43.8%)	33 (45.2%)
	NFL	20 (27.4%)	24 (32.9%)	29 (39.7%)
	NHL	45 (61.6%)	17 (23.3%)	11 (15.1%)

*NBA, National Basketball Association; NFL, National Football League; NHL, National Hockey League.*

The data were considered appropriate for model estimation using mediation. The distribution of the data was reasonably normal based on skewness and kurtosis less than 2 or greater than −2, indicating symmetrical distributions for variables (Pallant, 2020). Correlations were performed to characterize the relationships among variables (Pallant, 2020). As all variables were correlated for favorite and least favorite leagues, the aforementioned validity tests and the multidimensional scales were conceptually associated (Sony and Naik, 2012; Kopcha et al., 2014). Multicollinearity was not an issue as all correlations were below 0.85 (Berry et al., 1985; Bollen and Lennox, 1991). The detailed results of correlations for favorite and least favorite leagues are reported in Tables 3, 4, respectively.

**TABLE 3:** Rankings for favorite and least favorite league.

Variables	1	2	3	4	5
(1) FOVER	–				
(2) TOLER	0.35**	–			
(3) FCSR	0.26**	0.44**	–		
(4) FBA	0.21**	0.46**	0.73**	–	
(5) FBL	0.15*	0.40**	0.70**	0.84**	–
*M*	5.75	5.00	5.18	5.34	5.51
*SD*	1.96	1.35	1.10	0.91	0.91
Skewness	–0.24	–0.68	–0.83	–0.51	–0.97
Kurtosis	0.07	0.21	0.72	0.26	1.45
Cronbach’s α	0.70	.	0.89	0.92	0.89

*FOVER, favorite league overlap; TOLER, tolerance; FCSR, favorite CSR; FBA, favorite brand authenticity; FBL, favorite brand love.*

*In the case of tolerance, a single variable associated with multiculturalism was used since the variable does not have sufficient reliability.*

*p < 0.05; **p < 0.01.

**TABLE 4:** Bivariate relationships for least favorite league.

Variables	1	2	3	4	5
(1) LFOVER	–				
(2) TOLER	0.27**	–			
(3) LFCSR	0.37**	0.43**	–		
(4) LFBA	0.33**	0.45**	0.76**	–	
(5) LFBL	0.37**	0.40**	0.84**	0.75**	–
*M*	5.48	5.00	4.87	5.12	5.01
*SD*	1.98	1.35	1.27	0.89	1.15
Skewness	–0.25	–0.68	–0.99	–0.39	–0.99
Kurtosis	–0.17	0.21	0.69	0.09	1.08
Cronbach’s α	0.76	.	0.92	0.93	0.92

*LFOVER, least favorite league overlap; TOLER, tolerance; LFCSR, least favorite CSR; LFBA, least favorite brand authenticity; LFBL, least favorite brand love.*

*In the case of tolerance, a single variable associated with multiculturalism was used since the variable does not have sufficient reliability.*

**p < 0.01.

The estimated R-square values for the endogenous latent variables were analyzed to determine how much each variable influenced the four factors in each model. As shown in the pattern of correlations (see Tables 3, 4), the predictor and the three mediators have significant relationships with brand love. All paths for the mediation model and the corresponding coefficients, indirect effects, and bias-corrected 95% confidence intervals are displayed in Tables 5, 6.

**TABLE 5:** Model estimates for favorite league.

			95% CI	
Path	*β*	*SE*	LLCI	ULCI
**Direct effect**				
(1) FOVER → TOLER	0.348*	0.067	0.234	0.452
(2) FOVER → FCSR	0.124	0.065	0.017	0.228
(3) FOVER → FBA	–0.028	0.043	–0.099	0.041
(4) FOVER → FBL	–0.046	0.032	–0.103	0.003
(5) TOLER → FCSR	0.401*	0.072	0.276	0.512
(6) TOLER → FBA	0.180*	0.061	0.082	0.283
(7) TOLER → FBL	0.000	0.050	–0.082	0.083
(8) FCSR → FBA	0.662*	0.070	0.555	0.750
(9) FCSR → FBL	0.182*	0.071	0.070	0.309
(10) FBA → FBL	0.715*	0.070	0.587	0.822
**Indirect effects from FOVER to FBL**				
Total effect	0.152*	0.068	0.037	0.264
Total indirect effect	0.197*	0.063	0.092	0.298
(1) FOVER → TOLER → FBL	0.000	0.018	–0.029	0.030
(2) FOVER → FCSR → FBL	0.023*	0.015	0.005	0.055
(3) FOVER → FBA → FBL	–0.020	0.031	–0.073	0.029
(4) FOVER → TOLER → FCSR → FBL	0.025*	0.013	0.010	0.055
(5) FOVER → TOLER → FBA → FBL	0.045*	0.019	0.020	0.084
(6) FOVER → FCSR → FBA → FBL	0.059*	0.031	0.011	0.113
(7) FOVER → TOLER → FCSR → FBA → FBL	0.066*	0.019	0.041	0.107

*FOVER, favorite league overlap; TOLER, tolerance; FCSR, favorite CSR; FBA, favorite brand authenticity; FBL, favorite brand love.*

*In the case of tolerance, a single variable associated with multiculturalism was used since the variable does not have sufficient reliability.*

*Significant effect.

**TABLE 6:** Model estimates for least favorite league.

			95% CI	
Path	*β*	*SE*	LLCI	ULCI
**Direct effect**				
(1) LFOVER → TOLER	0.266*	0.064	0.160	0.370
(2) LFOVER → LFCSR	0.275*	0.063	0.172	0.380
(3) LFOVER → LFBA	0.041	0.051	–0.042	0.128
(4) LFOVER → LFBL	0.056	0.034	0.001	0.114
(5) TOLER → LFCSR	0.356*	0.077	0.221	0.477
(6) TOLER → LFBA	0.141*	0.055	0.050	0.233
(7) TOLER → LFBL	0.001	0.045	–0.70	0.080
(8) LFCSR → LFBA	0.688*	0.039	0.618	0.748
(9) LFCSR → LFBL	0.626*	0.069	0.512	0.733
(10) LFBA → LFBL	0.247*	0.076	0.125	0.369
**Indirect effects from LFOVER to LFBL**				
Total effect	0.370*	0.064	0.261	0.471
Total indirect effect	0.314*	0.055	0.218	0.401
(1) LFOVER → TOLER → LFBL	0.000	0.013	–0.019	0.022
(2) LFOVER → LFCSR → LFBL	0.172*	0.044	0.107	0.252
(3) LFOVER → LFBA → LFBL	0.010	0.013	–0.009	0.036
(4) LFOVER → TOLER → LFCSR → LFBL	0.059*	0.019	0.034	0.098
(5) LFOVER → TOLER → LFBA → LFBL	0.009*	0.006	0.003	0.023
(6) LFOVER → LFCSR → LFBA → LFBL	0.047*	0.018	0.023	0.082
(7) LFOVER → TOLER → LFCSR → LFBA → LFBL	0.016*	0.007	0.008	0.032

*LFOVER, least favorite league overlap; TOLER, tolerance; LFCSR, least favorite CSR; LFBA, least favorite brand authenticity; LFBL, least favorite brand love.*

*In the case of tolerance, a single variable associated with multiculturalism was used since the variable does not have sufficient reliability.*

*Significant effect.

### Favorite Leagues’ Identity Complexity and Their Brand Love

As shown in Table 5, brand authenticity in favorite leagues significantly influenced brand love (*β* = 0.715, *p* < 0.001), and CSR showed a significant impact on brand authenticity (*β* = 0.662, *p* < 0.001). Additionally, the total effect of identity complexity on brand love in favorite leagues was significant (*β* = 0.152, *p* = 0.025), and the total indirect effect of identity complexity on brand love was also significant after controlling for the three mediators (*β* = 0.197, *p* = 0.002). The indirect effects of identity complexity on brand love through CSR (H3), and the indirect effects of identity complexity on brand love through all mediators (H5) were significant. Specific indirect effects via tolerance and CSR, and tolerance and brand authenticity were also significant. These results for favorite leagues suggest that higher identity complexity predicts greater tolerance, which in turn leads to higher perceptions of CSR and brand authenticity, resulting in higher levels of brand love. The bootstrapped CIs indicated that the indirect effect of identity complexity on brand love through all mediators was the largest (*β* = 0.066) compared to those through CSR and brand authenticity (*β* = 0.059), tolerance and brand authenticity (*β* = 0.045), tolerance and CSR (*β* = 0.025), and CSR (*β* = 0.023).

### Least Favorite Leagues’ Identity Complexity and Their Brand Love

Among all direct paths, shown in Table 6, CSR showed significant effects on brand authenticity (*β* = 0.688, *p* < 0.001) and brand love (*β* = 0.626, *p* = 0.000); both the total effect (*β* = 0.370, *p* < 0.001) and the total indirect effect (*β* = 0.314, *p* < 0.001) were significant. Furthermore, the bias-corrected 95% CI indicated that the indirect effects of identity complexity on brand love through CSR, tolerance and CSR, CSR and brand authenticity, tolerance, CSR, and brand authenticity were significant. Comparisons showed that significant differences existed in the strength of the indirect path from identity complexity to brand love through each mediator: CSR (*β* = 0.172); tolerance and CSR (*β* = 0.059); CSR and brand authenticity (*β* = 0.047); tolerance, CSR, and brand authenticity (*β* = 0.016); and tolerance and brand authenticity (*β* = 0.009).

## Discussion

In this study, we examined the relationship between fans’ identity complexity and brand love via tolerance, CSR, and brand authenticity. Specifically, the mediating effects of tolerance, CSR, and brand authenticity in the association between identity complexity and brand love were investigated in relation to the degree of overlap among fans’ favorite and least favorite leagues. Results demonstrated that the relationship between social identity complexity and brand love is mediated by tolerance, CSR, and brand authenticity. Whereas identity complexity showed non-significant direct effects on brand love for both favorite or least favorite leagues, multiple mediators indicated positive influences via serial mediation involving all paths. The different results across favorite and least favorite league suggests that further research of identity complexity is needed, which is in line with suggestions that research on social identity complexity should examine the interplay among multiple identities on behavior (Roccas and Brewer, 2002; Brewer and Pierce, 2005; Kwon et al., 2015; Lee et al., 2020).

The results from this study illustrate three important points. First, although all variables were significantly associated (see Tables 3, 4), identity complexity based on fans’ favorite or least favorite league did not directly influence their brand love for the league when holding other variables constant. Second, all indirect effects including CSR as a mediator were significant. This infers that CSR has an imperative role in the causal chain with other variables, regardless of fans’ preferences. Third, the mediation effects showed significant results regardless of fans’ league preferences. This implies that identity complexity can lead to a positive view of a league, even if the league is least favorite, and expand the professional sport market.

The results of this study contribute to the existing literature on fan identification in sport marketing as it provides evidence of social identity complexity within sport fans who follow multiple professional sport leagues. Adding to Meyer (2014) who studied the relationship between fans’ multiple identifications and their tolerance, this study examined the associations of fans’ identity complexity with league brand, their tolerance, and the brand’s actions—CSR and brand authenticity—which influence perceptions of the league brand. This study also expands the framework of social identity complexity. Although Brewer and Pierce (2005) investigated in-group members’ perception of out-group members based on identity complexity and personality, this current study examined the attitudes of in-group members across their organizations that accept out-group members. In contrast to Brewer and Pierce’s findings that low overlap (high complexity) led to higher tolerance across multicultural groups based on White people’s perception of nationality, our results were consistent with Meyer’s study as high overlap (low complexity) led to higher tolerance across fans of different leagues.

This study contributes to the ongoing research on multiple identifications across different sport leagues. Due to perceptions of multiple identifications negatively affecting a primary identity (Putnam and Wondolleck, 2003), multiple identifications among sport fans have been largely neglected by scholars compared to studies involving single identification. However, it should be noted that scholars have started to focus on examining fans’ identity complexity (Meyer, 2014; Lee et al., 2020, 2021). The current study lays a foundation for more active research on identity complexity among sport fans. In the next section, we will provide insights into practical implications based on results from the current study.

## Practical Implications

This study provides information about developing both sport fans’ spectating cultures and the market size of professional sport leagues to marketing practitioners in the sport industry. These strategies can be divided into two main aspects: favorite league and least favorite league. With respect to the former, favorite leagues can increase revenue through marketing programs used by other leagues during their games. The current study also demonstrated that when fans have multiple identities related to professional sport leagues, their favorite league’s identity is not negatively influenced by other league identities. Furthermore, when the brand is associated with multicultural aspects, customers think of the brand more positively (Seo et al., 2015). For example, even though the NBA accepted advertisements that promoted other leagues’ schedules such as the NFL, the NHL, or MLB during NBA games, fans of the NBA did not experience an attenuation in their NBA identity (Heere and James, 2007; NBA, 2020). By using such methods, leagues may increase revenue and build positive relationships with other leagues. Furthermore, researchers should examine the role of identity complexity when there may be conflict situations across leagues (Lee et al., 2021).

Results from this study revealed that fans’ feelings about their least favorite leagues are more benevolent if fans have multiple identities. Therefore, from a long-term perspective, leagues should consider engaging in joint activities, such as initiating a CSR activity for a common cause that can overlap positive images across the leagues and deepen relationships with fans. On the other hand, in the case of least favorite league, organizations should work to eliminate negative images held by potential and current customers. As purchase intention is directly associated with brand image (Koronios et al., 2016), results from this study suggest that other leagues can adopt an effective method for changing fans’ attitudes.

Marketing practitioners should keep in mind that the relationship between fans and leagues depends on how fans evaluate CSR and brand authenticity. If a fan perceives a league’s CSR programs as authentic behavior, the fan’s brand love for their favorite or least favorite leagues will increase whether or not fans are tolerant about accepting other leagues’ cultures. Additionally, when the leagues continually share the detailed results of their CSR initiatives on a large-scale using media, fans will recognize the leagues’ behavior as credible and faithful. Through such announcements, fans may more highly evaluate not only the leagues’ CSR but also their brand authenticity. While these factors can affect relationships with fans, sport leagues should remember the important role of CSR and brand authenticity when developing marketing strategies.

## Limitations and Future Research

Although this study contributes to developing theoretical and practical implications in the field of sport management, there are some limitations that need to be resolved in future research. First, this study focused on only professional sport leagues in the US. To test the validity of this model, future researchers should study other countries (e.g., Australia, Canada, Germany, or South Korea) that operate multiple professional leagues. Additionally, as each country has different cultural passions associated with sport, future researchers should consider the unique characteristics of the country in their research. Second, this study used only one item on the questionnaire related to tolerance for multiculturalism due to issues with scale reliability. Since similar limitations have also existed in previous studies (e.g., Brewer and Pierce, 2005; Meyer, 2014), future research should employ other tolerance scales associated with social identity complexity. Third, common to survey research, there was no test of whether brand love is related to behavior. For instance, we did not measure whether higher brand love is related to buying more tickets or more merchandise. While we provide an initial first step in finding the associations among the constructs, future researchers should measure actual behaviors, or even intentions to purchase game tickets or merchandise at the least. In addition, researchers could design experimental manipulations of brand love to see whether increasing brand love increases consumption behaviors. Lastly, this study did not provide sufficient practical and theoretical recommendations for how leagues can induce their fans to establish multiple identities in conjunction with other leagues. The purpose of this study is to understand identity complexity in the sport industry; however, current results apply specifically to fans with multiple identities. Therefore, future researchers should develop a strategy for how people develop multiple identities in professional sport leagues.

## Conclusion

In conclusion, we looked into how fans’ multiple identifications with sports leagues affect multicultural tolerance and attitudes toward CSR initiatives. Moreover, the impact of CSR perception on perceived brand authenticity and brand love was investigated. The chain of effects is underscored as the serial mediation model was supported. The current study adds to the body of research on branding by emphasizing social identity complexity, tolerance, corporate social responsibility, and brand authenticity as antecedents of brand love. The perceived overlap among sports fans who identify with multiple leagues, social identity complexity, is highlighted as a personal disposition that influences the brand love perception process. Furthermore, the perception process for fans’ brand love for their favorite league against a less preferred league differed regarding the strength of indirect effects.

## Data Availability Statement

The raw data supporting the conclusions of this article will be made available by the authors, without undue reservation.

## Ethics Statement

The studies involving human participants were reviewed and approved by Texas A&M Institutional Review Board. The patients/participants provided their written informed consent to participate in this study.

## Author Contributions

All authors listed have made a substantial, direct, and intellectual contribution to this work, and approved the submitted version.

## Conflict of Interest

The authors declare that the research was conducted in the absence of any commercial or financial relationships that could be construed as a potential conflict of interest.

## Publisher’s Note

All claims expressed in this article are solely those of the authors and do not necessarily represent those of their affiliated organizations, or those of the publisher, the editors and the reviewers. Any product that may be evaluated in this article, or claim that may be made by its manufacturer, is not guaranteed or endorsed by the publisher.

## Appendix

**TABLE A1:** Measurement items.

Constructs and items	
*Social identity complexity*	NBA / NFL, NHL, and MLB NFL / NBA, NHL, and MLB NHL / NBA, NFL, and MLB MLB / NBA, NFL, and NHL
*Tolerance*	It is better for the country if racial and ethnic groups maintain their distinct customs and traditions
*CSR*	I am aware of the social programs of my favorite OOO
	I know of the good things my favorite OOO does for the community
	I believe my OOO to be a socially responsible organization
	I feel good about my favorite OOO partly because of all the things they do to benefit the community
	Part of the reason I like my favorite OOO is because of what they do for the community
	One of the reasons I speak positively about my favorite OOO is because of what they do for the community
	I buy merchandise from my favorite OOO partly because I believe they are a socially responsible organization
*Brand authenticity*	OOO is a brand with a history
	OOO is a timeless brand
	OOO is a brand that survives times
	OOO is a brand that survives trends
	OOO is a brand that will not betray you
	OOO is a brand that accomplishes its value promise
	OOO is a honest brand
	OOO is a brand that gives back to its consumers
	OOO is a brand with moral principles
	OOO is a brand true to a set of moral values
	OOO is a brand that cares about its consumers
	OOO is a brand that adds meaning to people’s lives
	OOO is a brand that reflects important values people care about
	OOO is a brand that connects people with their real selves
	OOO is a brand that connects people with what is really important
*Brand love*	OOO is a wonderful brand
	OOO makes me feel good
	OOO is totally awesome
	I have positive feelings about OOO
	OOO makes me very happy
	I love OOO!
	OOO is a pure delight

*OOO means participants’ favorite and least favorite professional sports leagues in the US based on the rank each league.*
